# Ischemic stroke prediction using machine learning in elderly Chinese population: The Rugao Longitudinal Ageing Study

**DOI:** 10.1002/brb3.3307

**Published:** 2023-11-07

**Authors:** Huai‐Wen Chang, Hui Zhang, Guo‐Ping Shi, Jiang‐Hong Guo, Xue‐Feng Chu, Zheng‐Dong Wang, Yin Yao, Xiao‐Feng Wang

**Affiliations:** ^1^ Department of Computational Biology, School of Life Sciences Fudan University Shanghai China; ^2^ Department of Cardiovascular Disease Aging Research Fudan University–the People's Hospital of Rugao Joint Research Institute of Longevity and Aging Rugao Jiangsu China; ^3^ Zhangjiang Fudan International Innovation Center, Human Phenome Institute Fudan University Shanghai China; ^4^ The People's Hospital of Rugao Rugao Jiangsu China

**Keywords:** ischemic stroke, logistic regression, machine learning, prediction, risk factors

## Abstract

**Objective:**

Compared logistic regression (LR) with machine learning (ML) models, to predict the risk of ischemic stroke in an elderly population in China.

**Methods:**

We applied 2208 records from the Rugao Longitudinal Ageing Study (RLAS) for ischemic stroke risk prediction assessment. Input variables included 103 phenotypes. For 3‐year ischemic stroke risk prediction, we compared the discrimination and calibration of LR model and ML methods, where ML methods include Random Forest (RF), Gaussian kernel Support Vector Machines (SVM), Multilayer perceptron (MLP), K‐Nearest Neighbors Algorithm (KNN), and Gradient Boosting Decision Tree (GBDT) to develop an ischemic stroke risk prediction model.

**Results:**

Age, pulse, waist circumference, education level, β2‐microglobulin, homocysteine, cystatin C, folate, free triiodothyronine, platelet distribution width, QT interval, and QTc interval were significant induced predictors of ischemic stroke. For ischemic stroke prediction, the ML approach was able to tap more biochemical and ECG‐related multidimensional phenotypic indicators compared to the LR model, which placed more importance on general demographic indicators. Compared to the LR model, SVM provided the best discrimination and calibration (C‐index: 0.79 vs. 0.71, 11.27% improvement in model utility), with the best performance in both validation and test data.

**Conclusion:**

In a comparison of LR with five ML models, the accuracy of ischemic stroke prediction was higher by combining ML with multiple phenotypes. Combined with other studies based on elderly populations in China, ML techniques, especially SVM, have shown good long‐term predictive performance, inspiring the potential value of ML use in clinical practice.

## Graphical abstract

Gaussian kernel Support Vector Machines (SVM) is an effective ML strategy for ischemic stroke risk prediction in a large population with a multidimensional phenotypic dataset.

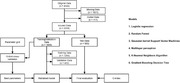

## 1 INTRODUCTION

Stroke is of great concern as one of the major diseases worldwide. In China, stroke is the leading cause of death and disability in adults, and the morbidity and mortality rates are stable or increasing. Some studies indicate that the prevalence of stroke in China (2.6% in 2020) is already higher than the estimated global prevalence of stroke (1.2% in 2019) (Tu et al., 2023). It has been reported that the incidence of stroke in China occurs mostly in the elderly (Bonita et al., 2004; Feigin et al., 2014; Wang et al., 2008; Yong‐Jun, 2010). As the major type of stroke, acute treatment of ischemic stroke is highly dependent on early prediction, and having timely treatment decisions is the cornerstone of acute stroke management (Wang et al., 2008).

In recent years, there has been a large body of research work related to stroke and its complications based on LR; for example, Johnston et al. (2007) validated and improved scores to predict very early stroke risk after transient ischemic attack by deriving new uniform scores based on LR. Lian et al. (2020) developed a risk prediction model by deriving LR for cohorts to develop early prediction of post‐ischemic stroke brain‐heart syndrome: the PANSCAN scale. Such risk scores are usually derived based on LR risk models and have been validated mainly in Europe and the United States. However, underestimation of stroke risk by such LR models in the contemporary Chinese elderly population due to poor self‐calibration may lead to inaccurate identification of individuals at high risk for ischemic stroke who would benefit from timely treatment, and new risk models should be developed for use in such populations (Chien et al., 2010; Leung et al., 2018; Xing et al., 2019).

In recent years, with the widespread use of big data, artificial intelligence has deduced excellent predictive value in predicting stroke risk with the excellent characteristics of ML in automating the decision‐making process (Hung et al., 2017; Khosla et al., 2010; Leung et al., 2018; Liu et al., 2019; Weng et al., 2017). For instance, Wu and Fang (2020) showed that the ML method with data balancing technique is an effective tool for stroke prediction using unbalanced data. Therefore, the construction of risk prediction models with more robust utility is the goal of our further work. To the best of our knowledge, previous AI‐based research efforts on stroke in Chinese elderly are interesting attempts but limited. The utility of stroke risk prediction models is poor and mostly based on small variables and small sample data, and little is known about the utility of such models for predictive assessment (Yong‐Jun, 2010).

The objectives of this study were to (i) identify important differential indicators between the ischemic stroke elderly population and healthy elderly population in China; (ii) develop and compare LR and ML models to identify significant predictors of induced ischemic stroke and to predict potential ischemic stroke episodes; and (iii) find optimal ML methods to decide in advance whether preventive interventions are needed to provide some diagnostic substantiate for clinical medicine.

## 2 MATERIALS AND METHODS

### 2.1 Study population

Data used in this study were obtained from the Rugao Longitudinal Ageing Study (RLAS). RLAS was designed to examine aging health trajectories and outcomes, and its design have been described elsewhere (Liu et al., 2016). This is a population‐based longitudinal study conducted in Rugao, Jiangsu Province, China, with data pooled for 2017 and 2019 results. The study was conducted in accordance with the Declaration of Helsinki and approved by the Human Ethics Committee of the School of Life Sciences, Fudan University. Informed consent (BE1815) was obtained from each participant.

Initially, 1872 and 2274 records of older adults aged 62–97 years were obtained from 31 communities in Jiang'an Township, Rugao City, in 2017 and 2019, respectively, based on age and gender strata at age 5 years. A follow‐up survey was conducted at 3 years to repeatedly measure baseline variables and to collect cross‐sectional data on prevalence. We excluded 1938 incomplete or abnormal records, and a total of 2208 records (2166 records of healthy participants and 42 records of participants with ischemic stroke) were used in the ischemic stroke prediction risk assessment study. Their eligible volunteers were diagnosed with ischemic stroke within 48 hours of symptom onset at a township health center or above, while excluding the possibility of having other diseases, such as cardiovascular or other neurovascular diseases. Data indicators are shown in Table S1 of the Supplementary Material and include questionnaires, demographics, traditional risk factors, electrocardiograms, and biochemistry.

### 2.2 Model development and evaluation

In this study, we downscaled the 30 indicators of the Simple Intelligence Mental State Examination Scale (MMSE) with the help of principal component analysis (PCA), and the specific methods and details are described in Method S1 of the Supplementary Material. Logistic regression (LR), Random Forest (RF), Gaussian kernel Support Vector Machines (SVM), Multilayer perceptron (MLP), K‐Nearest Neighbors Algorithm (KNN), and Gradient Boosting Decision Tree (GBDT) models were applied in the baseline survey obtained from the training set for ischemic stroke risk prediction in elderly people in Rugao city (Supplementary Material Method S3 for model details). Ten‐fold cross‐validation was used to select features in the model and adjust hyperparameters. Briefly, 10‐fold cross‐validation means that all data are divided into ten equal parts. Then, nine of these parts are simulated for training, and the rest are the test set (Kohavi, 1995; Wu & Fang, 2020). Finally, the average of the results is measured to obtain a more stable application. The detailed methods and results of parameter tuning are defined in Tables S3–S9 and Figure S1 in the Supplementary Material. The derivation and validation of the ML methods were done by Python 3.9 and the Scikit‐learn toolkit.

### 2.3 Statistical analysis

Continuous variables were expressed as the mean ± standard deviation and One‐Hot coding was applied to categorical variables. Wilcoxon rank‐sum test and 𝜒^2^ test were used for statistical comparisons to identify important indicators of variability between the elderly population with ischemic stroke and the healthy elderly population. Specific details are further elaborated in Supplementary Method S2. Two‐tailed *p* < .05 was appraised statistically significant.

**FIGURE 1:**
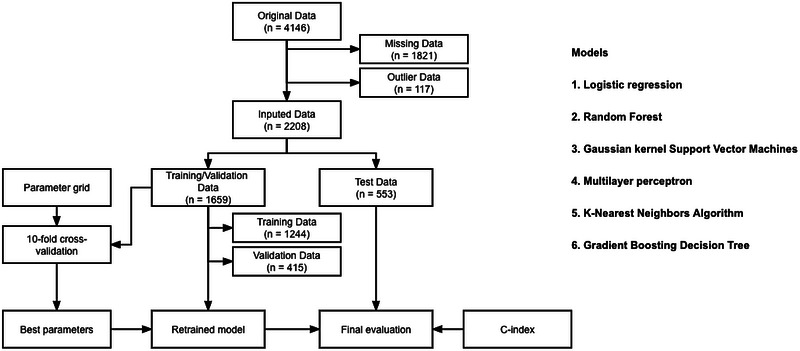
A flowchart describing the general framework of the study. Models were built using the training dataset, and the test dataset was used for computing the C‐index shown in Table 2.

Figure 1 derives the statistical analysis procedure followed in this study. Considering the resulting heavily imbalanced data samples, we made the LR and ML models learn as much as possible by giving higher weights to a few classes of samples (ischemic stroke class), setting the weight parameter “balanced” in the classifier, and lattice‐searching the hyperparameters with 10‐fold cross‐validation. Seventy‐five percent of the data set was stratified from the entire participant group for training/validation; the remaining 25% was used for testing.

## 3 RESULTS

A total of 2208 records were included. The mean age was 78.0 ± 4.4 years, of which 52.8% (*n* = 1166) were female and 43.6% (*n* = 963) were illiterate. Overall, 7.6% (*n* = 167) had symptoms of cerebral infarction in the past (medical history diagnosed at the township health center level or higher), 23.1% (*n* = 511) had a history of smoking either currently or for more than 6 consecutive months in the past, and 34.9% (*n* = 771) had a history of alcohol consumption either currently or for more than 6 consecutive months in the past. Table 1 derives the results of our Wilcoxon rank‐sum test (continuous indicators) and 𝜒^2^ test (subtype indicators) for the conventional data indicators, and the complete test results are described in Table S2 of the Supplementary Material. Under the hypothesis test two‐tailed *p* < .05, older adults with ischemic stroke were found to be older (79.6 vs. 77.9), to have wider waist circumference (95.2 vs. 90.3), lower education levels, more stable pulse, and to be more likely to have a history of cerebral infarction (47.6% vs. 6.7%) compared with the healthy elderly population. At the same time, cystatin C (1.2 vs. 1.1), folate (8.9 vs. 10.5), homocysteine (18.2 vs. 15.9), and β2‐microglobulin levels (2.5 vs. 2.3) in these ischemic stroke patients were statistically significantly different from the healthy elderly population.

**TABLE 1:** Characteristics of the elderly study population in Rugao (Conventional Indicators).

	Stroke (*n* = 42)	Non‐Stroke (*n* = 2166)	*p* Value
**Traditional risk factors, demographics, anthropometry, site**			
mean ± SD Age, y	79.57 ± 3.93	77.93 ± 4.41	<.05
Men, *n* (%)	23 (54.76%)	1023 (46.33%)	.41
Women, *n* (%)	19 (45.24%)	1147 (51.95%)	
mean ± SD Weight, kg	58.64 ± 9.15	58.33 ± 10.67	.81
mean ± SD Height, cm	155.39 ± 9.56	155.84 ± 9.37	.95
mean ± SD Waist, cm	95.24 ± 10.43	90.30 ± 10.23	<.05
mean ± SD Hip, cm	97.34 ± 10.21	95.71 ± 10.30	.35
mean ± SD Fasting GLU, mmol/L	6.02 ± 2.46	5.85 ± 1.48	.63
mean ± SD SBP, mmHg	126.18 ± 35.51	120.72 ± 36.18	.20
mean ± SD DBP, mmHg	86.73 ± 11.40	85.11 ± 11.06	.40
Pulse stability			
Stable	36 (85.71%)	2081 (94.25%)	<.05
Fairly stable	5 (11.90%)	58 (2.63%)	
Unstable	1 (2.38%)	31 (1.40%)	
**Questionnaire**
History of cerebral infarction			
Yes	20 (47.62%)	147 (6.66%)	<.05
No	22 (52.38%)	2023 (91.62%)	
Smoking condition			
No smoking	35 (83.33%)	1666 (75.45%)	.37
Currently smoking	6 (14.29%)	325 (14.72%)	
Have smoked for more than 6 months	1 (2.38%)	179 (8.11%)	
Drinking condition			
Not drinking	33 (78.57%)	1408 (63.77%)	.08
Currently drinking	9 (21.43%)	583 (26.4%)	
Drinking for more than 6 months	0 (0.00%)	179 (8.11%)	
Education level			
No schooling	19 (45.24%)	944 (42.75%)	<.05
Elementary school and above	21 (50.00%)	1216 (56.14%)	
Others	2 (4.76%)	10 (0.45%)	
**Biochemical indicators**			
mean ± SD Cystatin C, mg/L	1.21 ± 0.37	1.10 ± 0.32	<.05
mean ± SD Folic acid, nmol/L	8.88 ± 4.12	10.53 ± 4.80	<.05
mean ± SD hsCRP, mg/L	2.64 ± 4.20	2.55 ± 4.38	.33
mean ± SD HDLC, mmol/L	1.67 ± 0.37	1.77 ± 0.43	.13
mean ± SD LDLC, mmol/L	2.95 ± 0.79	3.03 ± 0.71	.29
mean ± SD TG, mmol/L	1.41 ± 0.91	1.46 ± 1.00	.78
mean ± SD UA, μmol / L	303.24 ± 96.28	290.57 ± 92.04	.40
mean ± SD β2‐microglobulin, mg/L	2.51 ± 0.74	2.31 ± 0.77	<.05

*Note*: *p* Value indicates the significance level of the hypothesis test (Wilcoxon rank‐sum test for numeric variables and 𝜒^2^ test for categorical variables, two‐tailed *p* value < .05 was considered statistically significant).

hsCRP, high‐sensitivity C‐reactive protein; GLU, blood glucose; HDLC, high‐density lipoprotein cholesterol; LDLC, low‐density lipoprotein cholesterol; TG, triglyceride; UA, uric acid; SBP, systolic blood pressure; DBP, diastolic blood pressure.

### 3.1 Comparisons of LR and ML models to predict risk of ischemic stroke

We use the training dataset to build models using different methods and optimize them to reduce prediction errors. These models are then tried on a test dataset to check model performance and determine the best predictor variables. Table 2 derives the results of LR and five ML model hyperparameter settings, as well as the C‐index in the test dataset.

**TABLE 2:** Validation score and test performance (both using Concordance index as evaluation criterion) for each of the LR and ML models.

Model type	Validation score (95% CI)	Test performance (95% CI)
LR	0.62 (0.61–0.63)	0.71 (0.70–0.72)
RF	0.70 (0.69–0.71)	0.72 (0.69–0.75)
SVM	0.72 (0.70–0.74)	**0.79 (0.77**–**0.81)**
MLP	0.74 (0.71–0.77)	0.73 (0.70–0.76)
KNN	0.55 (0.53–0.57)	0.54 (0.52–0.56)
GBDT	0.55 (0.52–0.58)	0.60 (0.59–0.61)

**FIGURE 2:**
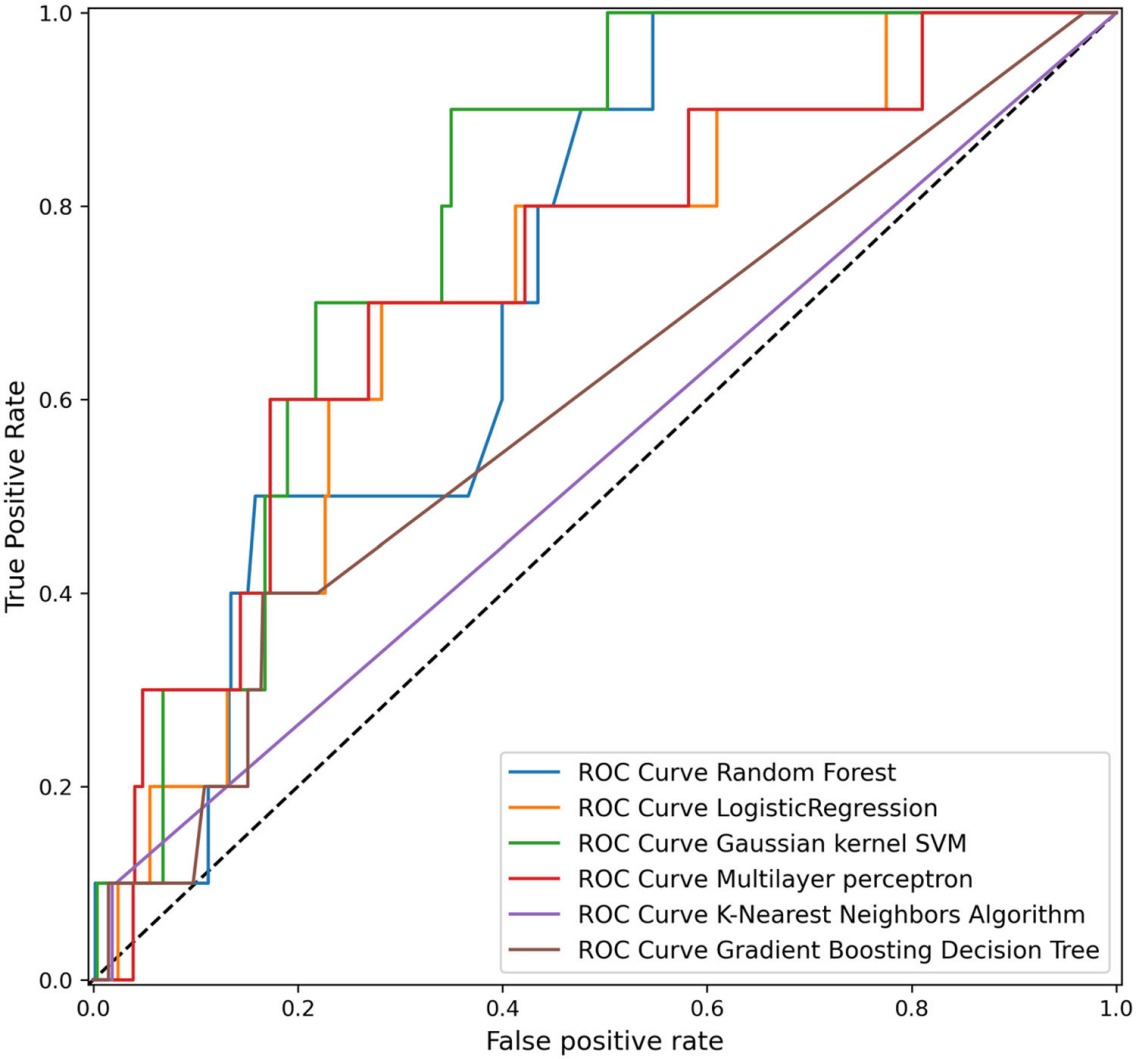
The receiver operating characteristic curves for LR and ML models in test data set. Receiver operating characteristic curves for the logistic regression (LR), Random Forest (RF), Gaussian kernel Support Vector Machines (SVM), Multilayer perceptron (MLP), K‐Nearest Neighbors Algorithm (KNN), and Gradient Boosting Decision Tree (GBDT) models in above. Each area under the curve indicates the corresponding C‐index of the model.

Overall, using the C‐index as an evaluation metric, some of the models derive a large improvement in performance on the test data compared to the validation scores, which indicates that the models have good generalization application and are of high practical value (Ambale‐Venkatesh et al., 2018; Harrell, 1982). Specifically, by plotting the Receiver operating characteristic (ROC) curve, as illustrated in Figure 2, we conclude that the performance varies widely among the models. The C‐index of SVM reaches a maximum value of 0.79 and is one of the best performing ML models. The C‐index of RF, MLP, and LR ranges from 0.71 to 0.73 and simulates well, while the application of KNN and GBDT is not satisfactory. Due to its high validation score and C‐index, SVM was confirmed as the best simulating model and was selected as a predictive ML model for ischemic stroke in the elderly population in China.

### 3.2 Selection of important predictors for the prediction model

Table 3 derives the top ten important predictor variables learned by LR, RF, and GBDT on the test dataset. These factors are the variables that play a key role in the models described above. Here, we show that the reason for choosing RF and GBDT in ML models is that for KNN, MLP and SVM, the inputs and outputs of the models can be intuitive, but determining the importance of features through the nonlinear model parameters behind them is very challenging and needs to be addressed.

**TABLE 3:** The top 10 important factors in the LR, RF, and GBDT methods.

Rank	Model type		
	LR	RF	GBDT
1	History of cerebral infarction	History of cerebral infarction	Education level
2	platelet distribution width	MMSE	History of cerebral infarction
3	Free triiodothyronine	Homocysteine	Cystatin C
4	Creatinine	SLDL‐CLDL‐cholesterol	Triglycerides
5	Weight	Folic acid	PR interval
6	Drinking condition	Triglycerides	Free triiodothyronine
7	Waist	Waist	QT interval
8	Albumin	Total cholesterol	QTc interval
9	Uric acid	Age	Lymphocytes
10	Systolic blood pressure	RV5 amplitude	Monocytes

*Note*: The relative variable importance of each variable can be assessed by visualizing the coefficients (which can be seen in Figure S2 in the Supplementary Material).

We observed that the older age of ischemic stroke patients in China may reflect the duration of risk exposure compared to the healthy elderly population, and that the wider waist circumference may be associated with irregular dietary habits and prolonged sedentary activity. On the other hand, lower education levels and a history of cerebral infarction appear to be significantly associated with an increased prevalence of ischemic stroke. Equally important, higher β2‐microglobulin was shown by biochemical markers to be more likely to lead to renal impairment, suggesting a high correlation between renal impairment and ischemic stroke. Similarly, higher levels of homocysteine and cystatin C can lead to atherosclerosis and thrombosis by damaging vascular endothelial cells and affecting lipid metabolism. Lower folate levels also lead to higher homocysteine, emphasizing the role of thrombosis as a common pathway leading to ischemic stroke. In addition to biomarkers with such a significant profile, lower free triiodothyronine and platelet distribution width also indicate the importance of hypothyroidism as well as inflammation for ischemic stroke. Finally, differences in QT interval (358.6 vs. 372.8) and QTc interval (397.8 vs. 412.7) on ECG suggest that increased risk of tachyarrhythmia is also a major predictor of ischemic stroke.

## 4 DISCUSSION

In this large prospective study of RLAS participants, we developed a new risk prediction model for predicting ischemic stroke in older adults in China. By comparing the analysis of LR with five ML models, we proposed a better SVM risk prediction model (11.27% improvement in C‐value compared with LR model). This SVM, which has a higher degree of discrimination relative to the LR, can translate into meaningful public health benefits. For example, a recent analysis of 100,000 British adults reported that a CVD polygenic risk score with a 0.012 increase in the C‐index could prevent 7% more CVD events than a traditional risk score alone (Sun et al., 2021). This model can tap more risk factors and hence more accurately make early risk prediction compared with previous prediction studies for Chinese elderly stroke, which is crucial for the treatment and prognosis of patients with ischemic stroke (Liu et al., 2007).

### 4.1 Logistic regression and machine learning

In contrast to previous risk prediction studies on ischemic stroke, we effectively evaluated the potential of ML techniques to improve risk prediction by comparing traditional LR‐based models with ML techniques for risk prediction. The results of this study suggest that ML methods are well suited for meaningful risk prediction in large‐scale epidemiological studies of extensive depth phenotypes. It is necessary to note that SVM‐based risk prediction methods provide the best event prediction compared to LR models. The wider range of predictors identified in our study compared to previous studies has inspired the possibility of ML methods in exploring frontier ischemic stroke risk factors based on multiple phenotypes. Significant calibration improvements in ML methods (such as SVM) relative to LR models are highly relevant to clinical practice, and currently, according to the Chinese Stroke Association's Executive Summary of Clinical Management of Ischemic Cerebrovascular Disease updated in 2019, decisions to initiate drug therapy can be made by defined risk thresholds, and barriers include the availability of data on certain risk factors and the need for regular updates and recalibration of clinical diagnostic guidelines (Liu et al., 2020). In this case, underestimation of stroke risk due to poor model calibration may result in failure to identify high‐risk individuals who could benefit from prophylactic drug therapy.

### 4.2 Methodological considerations

A major advantage of our models over previous stroke studies is that it is based on a prospective cohort of high‐quality longevity and aging data from Rugao, a typical representative city in China. We found that the sample characteristics of the Chinese aging population have their own specificity compared with those in Europe and the United States. Taking the California and ABCD scores constructed based on the LR model as an example, the LR model performed poorly in predicting the risk of ischemic stroke in the RLAS population while it performed better in Europe and the United States. Specifically, systolic blood pressure (SBP), diastolic blood pressure (DBP), and history of diabetes (HD) are included in the California and ABCD scores developed for European and American countries (Yong‐Jun, 2010). However, according to the Chinese RLAS cohort characteristics study, the proportion of SBP > 140 mm Hg in China was lower than that in clinic diagnoses in the United States (California clinic) and the United Kingdom (Oxford clinic) (36% vs. 60%, 36% vs. 48%), while the proportion of DBP > 90 mm Hg was higher (32% vs. 28%, 32% vs. 29%), and the proportion of HD was lower (8% vs. 18%, 8% vs. 10%). Therefore, blindly applying predictive models from previous studies will result in serious clinical diagnostic errors (Lian et al., 2020). On the other hand, compared to the LR model, traditionally, Mandrekar (2010) have suggested that with respect to subject working characteristic curves in diagnostic test assessment, a C‐value > 0.70 is considered acceptable and a value > 0.80 is excellent. Our constructed SVM has converging excellent predictive performance with a C‐index of almost 0.8 (95% CI, 0.77–0.81), which is relatively better than some of the previously constructed optimal ML risk prediction models (e.g., Ho et al., 2019, LR model C‐index: 0.765, Hilbert et al., 2019, Residual Neural Network model C‐index: 0.65, Van Os et al., 2018, LR model C‐index: 0.57) and can provide targeted guidance for ischemic stroke prevention and management in an older population aged 60 years and older in China.

On the other hand, in our training of supervised ML, we used a rigorous data cleaning method to ensure the quality of ischemic stroke data obtained from the RLAS cohort for training and testing, while taking into account the selection of variable features that are more readily available and have simpler and more convenient clinical utility than image data. Based on the aging difference in China, this risk prediction model construction for ischemic stroke in Chinese urban elderly is relevant and original, and it will be effective for subsequent advances in precision medicine (Benjamin et al., 2015; Cheng et al., 2014; Dawber et al., 1951).

Our study also has some limitations. Our study was conducted in the Chinese elderly cohort RLAS, and the SVM risk prediction model outlined in the study needs further external validation and refinement in clinical practice, in other aging populations in China, and potentially in other low‐ and middle‐income countries, as the RLAS cohort may not be representative of the entire Chinese elderly population or other populations.

## 5 CONCLUSIONS

Our prospective study manifests that for ischemic stroke prediction in the elderly population in China, the use of ML techniques improves risk prediction compared to traditional LR model approaches, with SVM providing the best discrimination and calibration performance. Older age, wider waist circumference, lower education level, kidney injury, atherosclerosis, thrombosis, hypothyroidism, and heart rate derangement were important predictors of predicting ischemic stroke. Our original research work provides some guidance for the application of big data based on multiple phenotypes. Through ML models construction, we will obtain more meaningful risk prediction, biomarker identification, and form data‐driven hypotheses.

## AUTHOR CONTRIBUTIONS

Huai‐Wen Chang: conceptualization; data analysis; and writing‐original draft preparation. Hui Zhang, Guo‐Ping Shi, Jiang‐Hong Guo, Xue‐Feng Chu, and Zheng‐Dong Wang: data collection and data cleaning. Xiao‐Feng Wang and Yin Yao: conceptualization and writing—reviewing and editing. All authors contributed to the article and approved the submitted version.

## CONFLICT OF INTEREST STATEMENT

None of the authors have any conflicts of interest in relation to this report.

### PEER REVIEW

The peer review history for this article is available at https://publons.com/publon/10.1002/brb3.3307.

## Supporting information


**Methods S1**. Principal component analysis.
**Methods S2**. Hypothetical test.
**Methods S3**. Details of logistic regression (LR) and machine learning (ML) model development
**Figures S1**. Ten‐fold cross‐validation grid search heatmap for model hyperparameter selection
**Figures S2**. Plots illustrating the variable importance for each of the 74 variables used in the analysis (in LR, RF and GBDT).
**Table S1**. A list of the markers that were used for prediction in this study.
**Table S2**. Characteristics of the elderly study population in Rugao.
**Table S3**. Ten‐fold cross‐validation grid search for ML model hyperparameter selection (using the C‐index as evaluation indicator).
**Table S4**. Hyperparameter selection in LR models.
**Table S5**. Hyperparameter selection in RF models.
**Table S6**. Hyperparameter selection in SVM models.
**Table S7**. Hyperparameter selection in MLP models.
**Table S8**. Hyperparameter selection in KNN models.
**Table S9**. Hyperparameter selection in GBDT models.

## Data Availability

The original contributions presented in the study are included in the article/supplementary material, further inquiries can be directed to the corresponding authors.
