# Supplementary material for: Ischemic stroke prediction using machine learning in elderly Chinese population: The Rugao Longitudinal Ageing Study
Source: Brain Behav. 2023 Nov 7;13(12):e3307. doi: 10.1002/brb3.3307 (PMC10726889; doi:10.1002/brb3.3307)
Supplement: Supplementary file 1 — Methods S1. Principal component analysis. Methods S2. Hypothetical test. Methods S3. Details of logistic regression (LR) and machine learning (ML) model development Figures S1. Ten‐fold cross‐validation grid search heatmap for model hyperparameter selection Figures S2. Plots illustrating the variable importance for each of the 74 variables used in the analysis (in LR, RF and GBDT). Table S1. A list of the markers that were used for prediction in this study. Table S2. Characteristics of the elderly study population in Rugao. Table S3. Ten‐fold cross‐validation grid search for ML model hyperparameter selection (using the C‐index as evaluation indicator). Table S4. Hyperparameter selection in LR models. Table S5. Hyperparameter selection in RF models. Table S6. Hyperparameter selection in SVM models. Table S7. Hyperparameter selection in MLP models. Table S8. Hyperparameter selection in KNN models. Table S9. Hyperparameter selection in GBDT models. [file BRB3-13-e3307-s001.docx]

**Supplementary Material for** **“Ischemic stroke prediction using machine learning in the elderly in Southern China: The Rugao Longevity and Aging Study”**

**Supplementary Methods S1.** Principal component analysis

Given a large number of clinical indicators, we employed principal component analysis (PCA) as a data reduction technique to simplify our modeling procedure while minimizing the loss of information. Thus, we employed PCA dimensionality reduction on 30 indicators of the Simple Intelligence Mental State Examination Scale (MMSE) in the training data. After mean-centering and standardizing each variable, PCA was applied. We selected principal components (PCs) with the criterion of a cumulative contribution rate greater than 95%. On the other hand, based on the idea of using a single indicator for the representation of the MMSE, we constructed 1-dimensional MMSE weighted indicators to replace the MMSE (containing 30 indicators) in the original data by weighting the contribution of the PCs retained after PCA for the 30 indicators in the MMSE.

**Supplementary Methods S2.** Hypothetical test

Wilcoxon rank-sum test

The basic idea of the Wilcoxon rank-sum test is as follows: if there is no difference between the two aggregates and the medians of the two samples of data selected are the same, the two samples of data will be mixed and sorted, and the two samples of data will be evenly distributed on the left and right sides of the medians so that the rank sum of each sample of data in the mixed arrangement will be similar. On the contrary, if the medians of the two samples are not the same, the rank sums of the two samples in the mixed arrangement will be more different after mixing and sorting the two sets of data. Therefore, the rank-sum corresponding to each of the two samples in the mixed data sort is the Wilcoxon rank-sum statistic. The two-sample rank-sum test was proposed by Wilcoxon as follows:

1. The hypothesis is proposed: $H_{0}:F\left( x \right)=G\left( y \right) H_{1}:F(x)\neq G(y)$.
2. Given the significance level *α*, single sample size *m*, *n*, and combined sample size *m + n*.
3. The rejection domain of$H_{0}$ is $T\leq r_{1}$,or $T\geq r_{2}$, where the critical value $r_{1}$ and$r_{2}$ are determined by the following equation: $P\{T\leq r_{1}\}=\frac{\alpha}{2}=P\{T\geq r_{2}\}$.
4. From the given ensemble sample values to find the rank sum of$X_{1},X_{2},......,X_{m}$ is $T=\sum_{i=1}^{m} R_{i}$. If $r_{1}<T<r_{2}$, then $H_{0}$ is accepted; otherwise, $H_{0}$ is rejected.

𝜒2 test

A chi-square test is a hypothesis test in which the distribution of a statistic approximately obeys the chi-square distribution when the null hypothesis holds. Its null hypothesis H0 is that the observed frequencies do not differ from the expected frequencies.

The basic idea of the 𝜒2 test is to first assume that $H_{0}$ holds, and based on this premise calculate the 𝜒2 value, which indicates the degree of deviation between the observed and the theoretical values. Based on the 𝜒2 distribution and degrees of freedom it is possible to determine the probability of obtaining the current statistic and more extreme cases in the case where the assumption of $H_{0}$ holds, P. If the value of P is small, it means that the observed value deviates too much from the theoretical value and the null hypothesis should be rejected, indicating a significant difference between the compared data; otherwise, the null hypothesis cannot be rejected and the actual situation represented by the sample cannot yet be considered to be different from the theoretical hypothesis are different.

**Supplementary Methods S3.** Details of logistic regression (LR) and machine learning (ML) model development

As shown in Figure S1, to obtain better generalization performance estimates, for each of the following six models, we used a 10-fold cross-validated grid search in the normalized training dataset to determine the optimal hyperparameter combinations based on the evaluation criteria of the C-index, and the results are shown in Table S3.

Logistic regression (LR) Models：

LR is a widely used linear classification model. For linear models used for regression, the output is a linear function of the features, being a line, plane, or hyperplane. For linear models for classification, the decision boundary is a linear function of the input. In other words, linear classifiers are classifiers that use straight lines, planes, or hyperplanes to separate two categories. There are many different algorithms for learning linear models, the difference being: how a particular combination of coefficients and intercepts is a good or bad measure of how well it fits the training data and whether regularization is used (or which regularization method is used).

Based on the above, we solve it with the help of Scikit-learn toolkit's LogisticRegression () function in Python 3.9, and the hyperparameter selection is shown in Table S4.

Random Forest (RF) Models:

We consider that decision trees often overfit the data and therefore consider the RF model as a solution to this problem. A random forest is essentially a collection of many decision trees, each of which is slightly different from the others. The idea behind random forests is that each tree makes relatively good predictions but may overfit some of the data. If many trees are constructed and each tree has good predictions, but all are overfitted in different ways, then we can average the results of these trees to reduce overfitting. This reduces overfitting while maintaining the predictive power of the trees, which can be proven mathematically rigorous.

Based on the above, we solve it with the help of Scikit-learn toolkit's RandomForestClassifier() function in Python 3.9, and the hyperparameter selection is shown in Table S5.

Gaussian kernel Support Vector Machines (SVM) Models：

Considering the linear indistinguishability of the data, we believe that kernel support vector machines (SVMs) are generalizable to extensions of more complex models that cannot be defined by the hyperplane of the input space. Fortunately, with the help of the kernel trick, it works by directly computing the inner product between data points in the extended feature representation without computing the extension and mapping the data into a higher-dimensional space. Combined with the nature of the data itself, we choose the radial basis function (RBF) kernel to implement the above idea. To achieve the prediction of ischemic stroke, the distance between its index data and each support vector needs to be measured, and the classification decision is made based on its distance from the support vector and the importance of the support vector learned during the training process.

Based on the above, we solve it with the help of Scikit-learn toolkit's SVC () function in Python 3.9, and the hyperparameter selection is shown in Table S6.

Multilayer perceptron (MLP) Models：

MLP can be considered as a generalized linear model that performs multi-layer processing to obtain the conclusion. In MLP, the process of computing the weighted summation is repeated several times, first computing the hidden units representing the intermediate processes, and then computing the weighted summation of these hidden units and obtaining the result. This model requires learning more weights: a coefficient between each input and each hidden unit, and a coefficient between each hidden unit and the output. After computing the weighted sum for each hidden cell, a nonlinear function is applied to the result, i.e., corrected nonlinearity (Relu), a tangent hyperbola (Tanh), or logarithmic odds (Logistic). The result of this function is then used in the weighted summation to calculate the output, which will allow the neural network to learn much more complex functions than the linear model.

Based on the above, we solve it with the help of Scikit-learn toolkit's MLPClassifier () function in Python 3.9, and the hyperparameter selection is shown in Table S7.

K-Nearest Neighbors Algorithm (KNN) Models：

As one of the simple machine learning algorithms, the KNN algorithm finds the nearest data point in the training data set, i.e., its "nearest neighbor", to predict a new data point. For any (k) neighbors, the labels are assigned using the "voting method". That is, for each test point, the number of neighbors belonging to category 0 and the number of neighbors belonging to category 1 is calculated, and the category with more occurrences (i.e., the category with the majority of k nearest neighbors) is used as the prediction result.

Based on the above, we solve it with the help of Scikit-learn toolkit's KNeighborsClassifier () function in Python 3.9, and the hyperparameter selection is shown in Table S8.

Gradient Boosting Decision Tree (GBDT) Models：

GBDT is an integrated learning method that constructs a more powerful model by merging multiple decision trees. Unlike RF, GBDT uses a sequential approach to constructing trees, where each tree tries to correct the errors of the previous one. By default, there is no randomization in GBDT, but rather strong pre pruning is used. GBDT usually uses trees of very small depth so that the model takes up less memory and predicts faster. the idea of GBDT is to merge many simple models, such as trees of small depth. Each tree can only make better predictions for some of the data, so adding more and more trees allows constant iteration to improve performance.

Based on the above, we solve it with the help of Scikit-learn toolkit's GradientBoostingClassifier () function in Python 3.9, and the hyperparameter selection is shown in Table S9.

**Supplementary Figures S1.** 10-fold cross-validation grid search heatmap for model hyperparameter selection

**
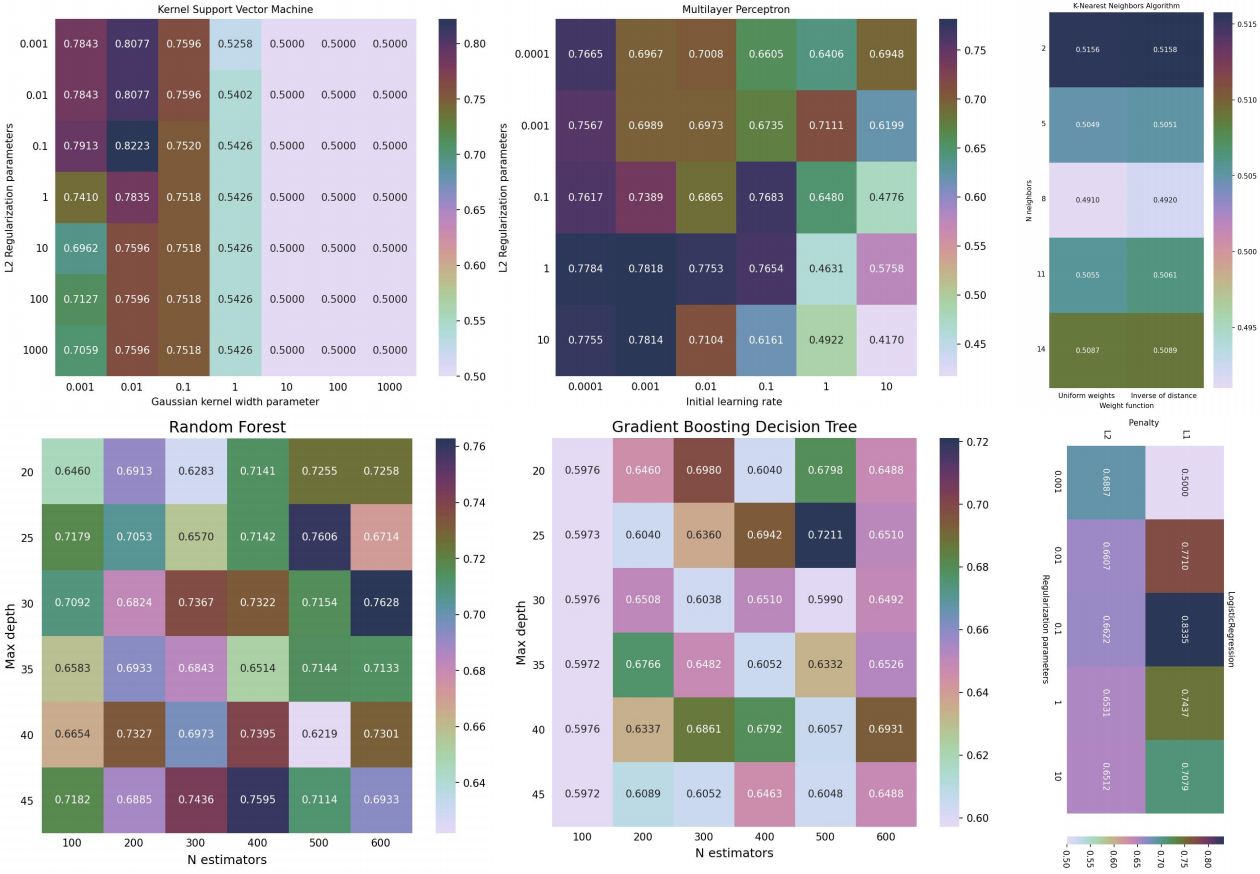
**

**Supplementary Figures S2.** Plots illustrating the variable importance for each of the 74 variables used in the analysis (in LR, RF and GBDT)


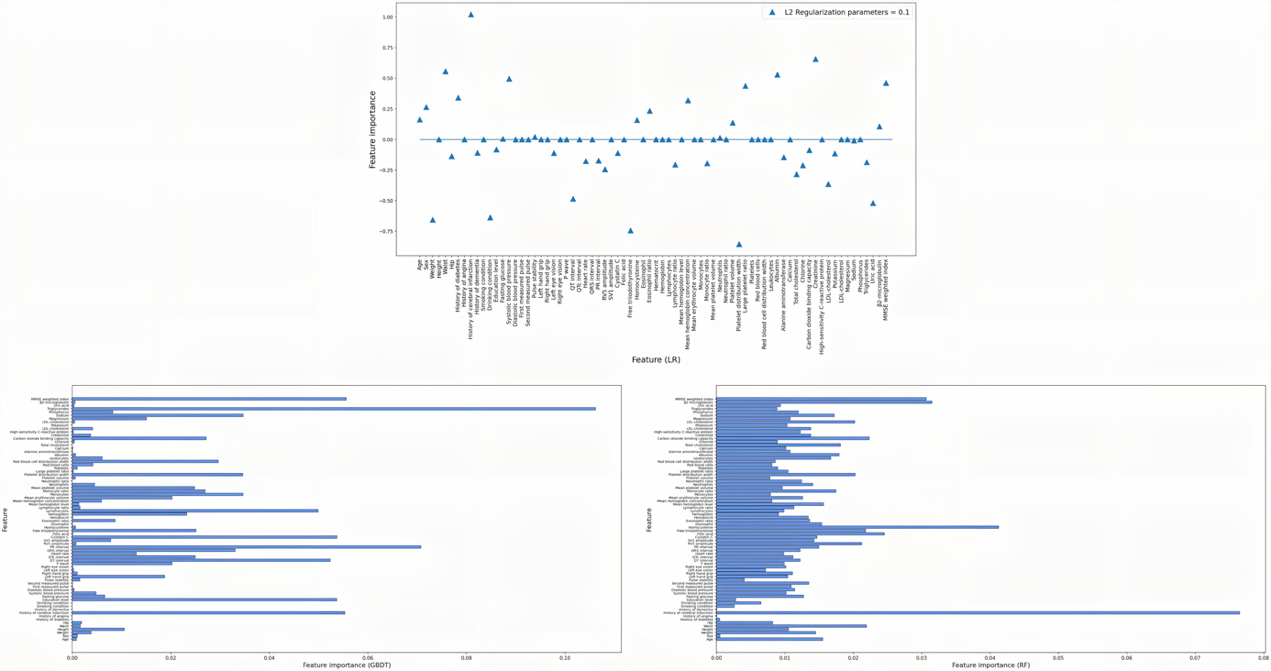


**Supplementary Table S1.** A List of the Markers That Were Used for Prediction in This Study

| **Traditional risk factors, demographics, anthropometry, site** |
| --- |
| Age, sex, weight, height, waist, hip, fasting glucose, systolic blood pressure, diastolic blood pressure, first measured pulse, second measured pulse, pulse stability, left hand grip, right hand grip, left eye vision, right eye vision |
| **Questionnaire** |
| History of diabetes, ﻿history of angina, ﻿history of cerebral infarction, ﻿history of dementia, smoking condition, drinking condition, education level, Mini-Intelligence Mental State Examination Scale (MMSE) |
| **Electrocardiographic main** |
| ﻿P wave, QT interval, QTc interval, Heart rate, QRS interval, PR interval, RV5 amplitude, SV1 amplitude |
| **Biochemical indicators** |
| ﻿Cystatin C, Folic acid, Free triiodothyronine, Homocysteine, Eosinophil, Eosinophil ratio, Hematocrit, Hemoglobin, Lymphocytes, Lymphocyte ratio, Mean hemoglobin level, Mean hemoglobin concentration, Mean erythrocyte volume, Monocytes, Monocyte ratio, Mean platelet volume, Neutrophils, Neutrophil ratio, Platelet volume, Platelet distribution width, Large platelet ratio, Platelets, Red blood cells, Red blood cell distribution width, Leukocytes, Albumin, Alanine aminotransferase, Calcium, Total cholesterol, Chlorine, Carbon dioxide binding capacity, Creatinine, High-sensitivity C-reactive protein, HDL-cholesterol, Potassium, LDL-cholesterol, Magnesium, Sodium, Phosphorus, Triglycerides, Uric acid, β2-microglobulin |

**Supplementary Table S2.** Characteristics of the Elderly Study Population in Rugao

|  | **Stroke**  **(n = 42)** | **Non-Stroke**  **(n = 2166)** | ***p-value*** |
| --- | --- | --- | --- |
| **Traditional risk factors, demographics, anthropometry, site** | | | |
| mean ± SD Age, y | 79.57 ± 3.93 | 77.93 ± 4.41 | ﻿0.01 |
| Men, n (%) | 23 (54.76%) | 1023 (46.33%) | ﻿0.41 |
| Women, n (%) | 19 (45.24%) | 1147 (51.95%) |  |
| mean ± SD Weight, kg | 58.64 ± 9.15 | 58.33 ± 10.67 | 0.81 |
| mean ± SD Height, cm | 155.39 ± 9.56 | 155.84 ± 9.37 | 0.95 |
| mean ± SD Waist, cm | 95.24 ± 10.43 | 90.30 ± 10.23 | 0.00 |
| mean ± SD Hip, cm | 97.34 ± 10.21 | 95.71 ± 10.30 | 0.35 |
| mean ± SD Fasting GLU, mmol/L | 6.02 ± 2.46 | 5.85 ± 1.48 | 0.63 |
| mean ± SD SBP, mmHg | 126.18 ± 35.51 | 120.72 ± 36.18 | 0.20 |
| mean ± SD DBP, mmHg | 86.73 ± 11.40 | 85.11 ± 11.06 | 0.40 |
| mean ± SD First measured pulse, times/min | 76.69 ± 15.60 | 76.30 ± 12.39 | 0.73 |
| mean ± SD Second measured pulse, times/min | 77.24 ± 14.52 | 76.54 ± 12.83 | 0.90 |
| Pulse stability |  |  |  |
| Stable | 36 (85.71%) | 2081 (94.25%) | ﻿0.00 |
| Fairly stable | 5 (11.90%) | 58 (2.63%) |  |
| Unstable | 1 (2.38%) | 31 (1.40%) |  |
| mean ± SD Left hand grip, kg | 19.62 ± 6.93 | 19.74 ± 7.19 | 0.95 |
| mean ± SD Right hand grip, kg | 20.33 ± 6.96 | 20.51 ± 7.38 | 0.82 |
| mean ± SD Left eye vision | 1.77 ± 2.01 | 1.91 ± 2.03 | 0.47 |
| mean ± SD Right eye vision | 1.78 ± 1.96 | 1.91 ± 2.03 | 0.54 |
| **Questionnaire** |  |  |  |
| History of diabetes |  |  |  |
| Yes | 5 (11.90%) | 173 (7.84%) | ﻿0.52 |
| No | 37 (88.10%) | 1997 (90.44%) |  |
| History of angina |  |  |  |
| Yes | 0 (0.00%) | 25 (1.13%) | ﻿1.0 |
| No | 42 (100.00%) | 2145 (97.15%) |  |
| History of cerebral infarction |  |  |  |
| Yes | 20 (47.62%) | 147 (6.66%) | ﻿0.00 |
| No | 22 (52.38%) | 2023 (91.62%) |  |
| History of dementia |  |  |  |
| Yes | 0 (0.00%) | 10 (0.45%) | ﻿1.0 |
| No | 42 (100.00%) | 2160 (97.83%) |  |
| Smoking condition |  |  |  |
| No smoking | 35 (83.33%) | 1666 (75.45%) | ﻿0.37 |
| Currently smoking | 6 (14.29%) | 325 (14.72%) |  |
| Have smoked for more than 6 months | 1 (2.38%) | 179 (8.11%) |  |
| Drinking condition |  |  |  |
| Not drinking | 33 (78.57%) | 1408 (63.77%) | ﻿0.08 |
| Currently drinking | 9 (21.43%) | 583 (26.4%) |  |
| Drinking for more than 6 months | 0 (0.00%) | 179 (8.11%) |  |
| Education level |  |  |  |
| No schooling | 19 (45.24%) | 944 (42.75%) | ﻿0.01 |
| Elementary school and above | 21 (50.00%) | 1216 (56.14%) |  |
| Others | 2 (4.76%) | 10 (0.45%) |  |
| **Electrocardiographic main** | | | |
| mean ± SD P wave, ms | 91.10 ± 14.74 | 91.91 ± 12.40 | 0.82 |
| mean ± SD QT interval, ms | 358.62 ± 63.47 | 372.77 ± 39.30 | 0.33 |
| mean ± SD QTc interval, ms | 397.76 ± 71.37 | 412.71 ± 46.28 | 0.56 |
| mean ± SD Heart rate, bpm | 71.38 ± 14.51 | 72.12 ± 12.62 | 0.58 |
| mean ± SD QRS interval, ms | 92.05 ± 17.95 | 89.55 ± 16.93 | 0.27 |
| mean ± SD PR interval, ms | 152.90 ± 22.11 | 157.05 ± 22.40 | 0.19 |
| mean ± SD RV5 amplitude, mv | 1.46 ± 0.58 | 1.58 ± 0.71 | 0.22 |
| mean ± SD SV1 amplitude, mv | 0.71 ± 0.53 | 0.73 ± 0.49 | 0.53 |
| **Biochemical indicators** |  |  |  |
| mean ± SD Cystatin C, mg/L | 1.21 ± 0.37 | 1.10 ± 0.32 | 0.04 |
| mean ± SD Folic acid, nmol/L | 8.88 ± 4.12 | 10.53 ± 4.80 | 0.04 |
| mean ± SD Free triiodothyronine, pmol/L | 4.83 ± 0.62 | 4.98 ± 0.68 | 0.24 |
| mean ± SD Homocysteine, μmol/L | 18.18 ± 6.75 | 15.92 ± 6.60 | 0.00 |
| mean ± SD Eosinophil | 0.13 ± 0.13 | 0.16 ± 0.15 | 0.14 |
| mean ± SD Eosinophil ratio | 2.17 ± 2.50 | 2.60 ± 2.28 | 0.06 |
| mean ± SD Hematocrit | 0.43 ± 0.04 | 0.43 ± 0.04 | 0.95 |
| mean ± SD Hemoglobin, g/L | 139.45 ± 14.31 | 138.33 ± 13.76 | 0.66 |
| mean ± SD Lymphocytes | 2.04 ± 0.66 | 2.07 ± 0.72 | 0.74 |
| mean ± SD Lymphocyte ratio | 31.72 ± 8.71 | 33.26 ± 8.92 | 0.27 |
| mean ± SD Mean hemoglobin level, pg | 31.51 ± 1.63 | 31.36 ± 1.75 | 0.79 |
| mean ± SD Mean hemoglobin concentration, g/L | 326.83 ± 12.47 | 323.98 ± 12.01 | 0.22 |
| mean ± SD Mean erythrocyte volume, fL | 96.48 ± 4.67 | 96.85 ± 5.31 | 0.48 |
| mean ± SD Monocytes | 0.48 ± 0.16 | 0.48 ± 0.17 | 0.84 |
| mean ± SD Monocyte ratio | 7.35 ± 1.69 | 7.68 ± 2.07 | 0.72 |
| mean ± SD Mean platelet volume, fL | 10.25 ± 4.42 | 10.57 ± 3.96 | 0.98 |
| mean ± SD Neutrophils | 3.90 ± 1.32 | 3.58 ± 1.26 | 0.10 |
| mean ± SD Neutrophil ratio | 58.66 ± 9.38 | 56.29 ± 9.32 | 0.13 |
| mean ± SD Platelet volume | 0.21 ± 0.10 | 0.21 ± 0.09 | 0.94 |
| mean ± SD Platelet distribution width, % | 12.32 ± 7.57 | 13.74 ± 6.15 | 0.71 |
| mean ± SD Large platelet ratio, % | 34.74 ± 17.66 | 35.54 ± 16.18 | 0.99 |
| mean ± SD Platelets | 191.67 ± 57.43 | 194.01 ± 63.74 | 0.83 |
| mean ± SD Red blood cells | 4.43 ± 0.42 | 4.42 ± 0.41 | 0.74 |
| mean ± SD Red blood cell distribution width, % | 13.59 ± 0.83 | 13.50 ± 0.88 | 0.42 |
| mean ± SD Leukocytes | 6.56 ± 1.60 | 6.29 ± 1.67 | 0.20 |
| mean ± SD Albumin, g/L | 45.37 ± 2.46 | 44.99 ± 2.47 | 0.34 |
| mean ± SD Alanine aminotransferase, U/L | 18.62 ± 8.53 | 19.31 ± 10.82 | 0.67 |
| mean ± SD Calcium, mmol/L | 2.38 ± 0.11 | 2.37 ± 0.12 | 0.64 |
| mean ± SD Total cholesterol, mmol/L | 5.18 ± 1.02 | 5.36 ± 0.97 | 0.16 |
| mean ± SD Chlorine, mmol/L | 101.63 ± 3.62 | 101.65 ± 3.48 | 0.85 |
| mean ± SD Carbon dioxide binding capacity, mmol/L | 27.39 ± 2.76 | 27.70 ± 2.15 | 0.64 |
| mean ± SD Creatinine, umol/L | 73.05 ± 21.90 | 67.05 ± 18.18 | 0.09 |
| mean ± SD hsCRP, mg/L | 2.64 ± 4.20 | 2.55 ± 4.38 | 0.33 |
| mean ± SD HDLC, mmol/L | 1.67 ± 0.37 | 1.77 ± 0.43 | 0.13 |
| mean ± SD Potassium, mmol/L | 4.67 ± 0.46 | 4.68 ± 0.48 | 0.98 |
| mean ± SD LDLC, mmol/L | 2.95 ± 0.79 | 3.03 ± 0.71 | 0.29 |
| mean ± SD Magnesium, mmol/L | 0.97 ± 0.09 | 0.96 ± 0.08 | 0.49 |
| mean ± SD Sodium, mmol/L | 140.47 ± 2.67 | 140.78 ± 2.12 | 0.48 |
| mean ± SD Phosphorus, mmol/L | 1.15 ± 0.15 | 1.14 ± 0.17 | 0.72 |
| mean ± SD TG, mmol/L | 1.41 ± 0.91 | 1.46 ± 1.00 | 0.78 |
| mean ± SD UA, μmol / L | 303.24 ± 96.28 | 290.57 ± 92.04 | 0.40 |
| mean ± SD β2-microglobulin, mg/L | 2.51 ± 0.74 | 2.31 ± 0.77 | 0.04 |

hsCRP indicates high-sensitivity C-reactive protein; GLU, blood glucose; HDLC, high-density lipoprotein cholesterol; LDLC, low-density lipoprotein cholesterol; TG, triglyceride; UA, uric acid; SBP, systolic blood pressure; and DBP, diastolic blood pressure. p-value indicates the significance level of the hypothesis test (Wilcoxon rank sum test for numeric variables and 𝜒2 test for categorical variables).

**Supplementary Table S3.** 10-fold cross-validation grid search for ML model hyperparameter selection (using the C index as evaluation indicator)

| **Model Type** | **Test set score** | **Best cross-validation score** |
| --- | --- | --- |
| LR | 0.6235 | 0.8335 |
| RF | 0.7317 | 0.7628 |
| SVM | 0.7233 | 0.8223 |
| MLP | 0.7405 | 0.7818 |
| KNN | 0.5541 | 0.5158 |
| GBDT | 0.5468 | 0.7211 |

**Supplementary Table S4.** Hyperparameter Selection in LR Models

| **Parameter** | **Select interval** | **Best value** |
| --- | --- | --- |
| penalty | ['l1', 'l2'] | 'l1' |
| C | [ 0.001,0.01,0.1,1,10] | 0.01 |
| class_weight | / | "balanced" |
| solver | / | 'liblinear' |

**Supplementary Table S5.** Hyperparameter Selection in RF Models

| **Parameter** | **Select interval** | **Best value** |
| --- | --- | --- |
| n_estimators | [100,200,300,400,500,600] | 600 |
| max_depth | [20,25,30,35,40,45] | 30 |
| min_samples_leaf | / | 1 |
| oob_score | / | True |
| class_weight | / | "balanced" |

**Supplementary Table S6.** Hyperparameter Selection in SVM Models

| **Parameter** | **Select interval** | **Best value** |
| --- | --- | --- |
| C | [ 0.001,0.01,0.1,1,10,100,1000] | 0.1 |
| gamma | [ 0.001,0.01,0.1,1,10,100,1000] | 0.01 |
| kernel | / | 'rbf' |
| class_weight | / | "balanced" |

**Supplementary Table S7.** Hyperparameter Selection in MLP Models

| **Parameter** | **Select interval** | **Best value** |
| --- | --- | --- |
| alpha | [0.0001,0.001,0.1,1,10] | 1 |
| learning_rate_init | [0.0001,0.001,0.01,0.1,1,10] | 0.001 |
| activation | / | 'logistic' |
| solver | / | 'adam' |
| max_ite | / | 10000 |

**Supplementary Table S8.** Hyperparameter Selection in KNN Models

| **Parameter** | **Select interval** | **Best value** |
| --- | --- | --- |
| n_neighbors | [2,5,8,11,14] | 2 |
| weights | [ 'uniform','distance'] | 'distance' |

**Supplementary** **Table S9.** Hyperparameter Selection in GBDT Models

| **Parameter** | **Select interval** | **Best value** |
| --- | --- | --- |
| n_estimators | [100,200,300,400,500,600] | 500 |
| max_depth | [20,25,30,35,40,45] | 25 |
| min_samples_leaf | / | 1 |
